# Mental Health Act (1987): Need for a paradigm shift from custodial to community care

**Published:** 2011-03

**Authors:** Suresh Bada Math, Pratima Murthy, Channapatna R. Chandrashekar

**Affiliations:** Department of Psychiatry, National Institute of Mental Health & Neuro Sciences (Deemed University), Bangalore 560 029, India

People with mental disorders are particularly vulnerable to abuse and violation of their rights^1^. If a protective mechanism is not in place, they are susceptible to abuse by anyone in the society^2^. In India, mental health care is not perceived as an important aspect of public health care. Hence, mental health legislation will play a very important role in upholding the rights of the mentally ill^2^,^3^. The fundamental aim of mental health legislation is to protect, promote and improve the lives and mental well-being of citizens.

Mental health legislations were initially drafted to safeguard the public from dangerous patients by isolating them from the public. A paradigm shift from custodial care to community care has occurred due to (*i*) Advances in medical technology in assessment and treatment of mental disorders; (*ii*) the human rights movement; (*iii*) World Health Organization’s (WHO) definition of ‘health’^4^ ; and (*iv*) Promotive, preventive, curative, rehabilitative approaches and mitigation of disability. This shift has given a new perspective to the care of mental disorders and has led to the review of mental health legislations worldwide^4^. Discrimination and stigma may impact access to adequate treatment and care as well as other areas of life, including employment, education, marriage and shelter. The inability to integrate into society as a consequence of these limitations can increase the isolation experienced by an individual, which can, in turn, aggravate mental disorder.

*Need for amendments in the Mental Health Act 1987 (MHA 1987):* The amendment of the MHA (1987) is considered very critical at this point of time because of two landmark developments. At the national level most exemplary amendments into the Protection of Human Rights Act of 1993 and the definition of ‘Human Rights’ and ‘International covenants’ have led to the broader concept of human rights which is enforceable in Indian judiciary^5^. At the International level, the most wanted ratification of the Convention on Rights of Persons with Disability in October 2007^6^ has further strengthened the need for amendments in MHA 1987.

MHA (1987) came into force in 1993, replacing the Indian Lunacy Act, 1912. MHA (1987) is divided into 10 chapters consisting of 98 sections. Some of the shortcomings and possible remedies are presented here.

*Rights of patients with mental illness:* Rights of patients with mental illness undermined can be brought forward by adding rights of the persons with mental illness in the preamble of MHA (1987). However, these rights should be balanced with the rights of the family because the primary care givers in India are the family, not the State. In India with inadequate resources for mental illness^8^, views and caregivers’ burden should also be kept in mind.

*Right to health:* Right to health for people with mental disorders means availability of mental health services, accessibility to the services and quality services with regard to both physical and mental health care. This can be achieved only through implementation of National Mental Health Programme. The emphasis thus needs to shift from ‘respect’ ‘promote’ and ‘protect’ to focus more on ‘fulfill’.

*Definition of mental illness:* It is necessary to redefine mental illness in the context of the Act to exclude mental retardation from the purview of the act as there are large numbers of individuals with mental retardation requiring psychiatric interventions^9^.

*Institutions under the MHA 1987:* The Constitution of India guarantees certain Fundamental Rights to all its citizens^10^.

Article 21, Right to life and liberty lays down that no person shall be deprived of his life or personal liberty except according to ‘procedure established by law’^11^. Hence, in any institution where Right to life and liberty are violated by putting the mentally ill in a closed ward, this amounts to violation of fundamental rights of a citizen if there is no ‘procedure established by law’. It is considered a serious crime and is punishable under the Indian Penal Code Sec 340, Sec 342, Sec 343 and Sec 344^12^. All these sections deal with wrongful confinement of any person. Any person found guilty under the above section invites punishment for a term, extending to three years or fine or both depending upon the number of days of wrongful confinement^12^. However, the fundamental rights embodied in Part III of the Constitution are not absolute but relative subject to public safety and security of the State. The restrictions may be imposed on the fundamental rights only in pursuance of law and restriction must not be arbitrary, unfair or unreasonable^10^,^11^. Deprivation of Article 21 (Right to life and personal liberty) can only be practiced under the ‘procedure established by law’. Procedure prescribed by law must be strictly followed and the law must be just, fair and reasonable^13^,^14^. Hence, those institutions where patients with mental illness are kept inside a closed ward or between four walls need to be under the purview of the Mental Health Act.

In the current MHA (1987) the review processes or appeal processes for mentally ill patients are far from realistic. Hence, it can be said that the procedure prescribed by the MHA (1987) for involuntary admission and treatment can be considered as arbitrary and unreasonable. This is a serious drawback of the Act. Hence, the appeal and review process requires to be reformulated so that it is simple, fair, just, reasonable and easily accessible to patients with mental illness inside the premises of the custodial care institutions.

*Definition of ‘psychiatric hospital’ or ‘psychiatric nursing home’:* The definition of various institutions is grouped together. This includes Convalescent homes^7^. Licensing requirements of the ‘psychiatric hospital’ is, one psychiatrist for every ten inpatient beds which is simply far from the ground reality. Hence, this requires to be made realistic by making it one psychiatrist for 100 inpatient beds. If the above definition and minimum standards are not amended then following are the implications and setbacks.

(*i*) All the mental health care centers such as Mental hospitals, Psychiatric Nursing Homes, Private General Hospital Psychiatry centres and Convalescent homes are grouped together in the Act. The minimum standards applicable to psychiatric hospitals which deal with acutely disturbed patients with severe illness also apply to convalescent homes, where the focus is on rehabilitation and reintegration into the community. Similar to Convalescent homes, De-addiction centres also require to be defined and also minimum standards to be formulated keeping the ground reality of available resources in mind.

(*ii*) Government mental hospitals were exempted from taking a license. This was a serious aberration in the MHA (1987). All government psychiatric hospitals should obtain license so that minimum standards are met as per the Act. Otherwise the very essence of the Act to protect the rights of patients with mental illness will be lost and the State will be indulging in the violation of the human rights of such persons in Government Mental Hospitals^15^. Licensing should be applicable to any place where *involuntary* treatment is offered in a closed setting, where the fundamental rights of the Indian constitution such as right to life and liberty are abrogated.

(*iii*) Only Psychiatry units in government general hospitals were kept out of the purview of the Act. Private general hospitals are finding it difficult to procure license and to meet the minimum norms of MHA 1987. In the spirit of the National Mental Health Programme, it is necessary to encourage voluntary treatment in general hospital settings^16^,^17^. Hence, both the government and private general hospital settings should be kept out of the purview of the Act.

(*iv*) A majority of the Indian population still seeks help from faith-healers, complementary and alternative medicines and religious centers^17^. Any settings which involves involuntary treatment should be brought under the purview of the Act to avoid tragedies like Erwadi^18^. The main essence of the Act is to protect the rights of the mentally ill patients in any setting. Similarly, patients with mental illness in any custodial care such as prison, juvenile home, home for mentally retarded, reception centres, working women hostels, *etc*., require to be monitored under the modified Act. There are instances in which patients were languishing in the mental institute for more than five decades and were able to get freed only after the intervention from the Honorable Supreme Court of India^19^.

*Psychiatric Emergency Services:* Unfortunately, there are no guidelines or provisions under MHA (1987) for emergency crisis intervention to help families caring for a mentally ill family member. The Supreme Court of India has stated that every doctor, whether at a government hospital or otherwise has the professional obligation to render medical services when it is required during an emergency situation with due expertise for protecting life^20^. Further, it has also accepted the right to health as a fundamental right^21^,^22^. Sometimes helpless family members are forced to file complaints against the mentally ill individual for petty crimes like violence, assault, property destruction, theft, robbery and so forth. Under such circumstances, law enforcing agencies file an FIR for the petty crime, arrest the mentally ill person and send them to judicial custody for many years without any treatment^23^. Obtaining a reception order is very difficult in the current MHA 1987. Hence the process of obtaining a reception order requires to be simplified by removing the judicial involvement in the process by formulating tribunals or hospitals boards or review committees such as Child Welfare Committee (CWC) of Juvenile Justice Act. Admission of wandering mentally ill patients also requires to be streamlined. Police personnel need to be sensitized and made accountable for facilitating admissions of such patients to the hospital under the Act.

*Choice of treatment:* MHA (1987) is silent regarding the consent for treatment, and the method to be adopted when a severely ill patient refuses well established treatments like medication or modified electroconvulsive therapy (ECT). Some hospitals have evolved standardized protocols for patients unable to provide consent. One method is to obtain the opinion of two psychiatrists independently and also the consent of the hospital RMO or superintendent who acts as a surrogate guardian^2^. The debate of modified ECT vs unmodified ECT should end because the palatability of the treatment gains importance from a human rights perspective. Hence, modified ECTs should be mandated in the new MHA. Research on mentally ill patients should follow ICMR ethical guidelines stringently^24^. Forced treatment should be distinguished from involuntary admission. Forced treatment requires to be defined and procedure requires to be outlined.

*Media and mentally ill persons:* Media including television, cinema and newspapers use mental illness as a means of publicity, sensationalism or misplaced humour. Such depictions continue to contribute to stigma and negative attitudes among the public. There is no provision to take action against such human right violations of the mentally ill^2^.

*Certificates and mentally ill persons:* There are many instances when family members of the mentally ill patients misuse the medical certificates for their benefit and have on occasion harassed the doctors for certificate under the Right to Information Act. Unfortunately, the concerned patient’s rights are violated during the whole process. Certificates need to be issued only to the patients after they recover from illness. In case of treatment resistant or refractory cases, certificates need to be issued to the family members only through a medical board. The board has to ensure adequate safeguards to protect the mentally ill against misuse of the certificate by the family members.

*Mental Health Authority:* Even after two decades of the MHA 1987, there are only five effectively functioning State Mental Health Authorities in the country. Main reason for non implementation is lack of resources. Hence, a yearly budget should be sanctioned for both Central and State Mental Health Authority for their smooth functioning. The parliamentary committee of the Indian Psychiatric Society has tried to bring amendments to the act but has not been successful so far. The user groups and non governmental organizations need to be represented in the Mental Health Authority both at Central and State level. During the review of this publication there have been major discussion on the amendment and a draft for a new Mental Health Care Act is under review.

In conclusion, there is an urgent need for amending the mental health legislation to meet the international and national obligations by the State towards its citizens. The rights of the persons with mental illness should be protected. However, these rights should be balanced with the rights of the family because the primary caregivers in India are the family and not the State. Planned amendments need to keep the ground reality of available resources and minimum standards needs to be formulated. Budget allocation for the mental health authorities is very essential for their effective functioning.
